# Efficacy and integration of different control methods against *Ostrinia nubilalis* Hübner (Lepidoptera: Crambidae) and *Sesamia nonagrioides* Lefebvre (Lepidoptera: Noctuidae) in Çukurova, Turkey

**DOI:** 10.1093/jisesa/ieag118

**Published:** 2026-11-05

**Authors:** Alican Kurtuluş, Serpil Kornoşor

**Affiliations:** Department of Plant Protection, Faculty of Agriculture, University of Çukurova, Adana, Turkey; Department of Plant Protection, Faculty of Agriculture, University of Çukurova, Adana, Turkey

**Keywords:** biological control, semiochemicals, integrated pest management

## Abstract

Lepidopteran stem borers—the European corn borer, *Ostrinia nubilalis* Hübner (Lepidoptera: Crambidae), and the Mediterranean corn borer, *Sesamia nonagrioides* Lefèbvre (Lepidoptera: Noctuidae)—cause substantial yield losses in maize production across the Mediterranean basin, yet reliance on broad-spectrum insecticides, particularly pyrethroids, in this system suppresses natural enemy activity and limits the development of sustainable integrated pest management (IPM) programs. A two-year field study (2012–2013) was conducted in Mersin province, Çukurova, Turkey, to compare the efficacy of four management strategies: integrated management (IM; mating disruption [MD] combined with inundative releases of *Trichogramma evanescens* Westwood [Hymenoptera: Trichogrammatidae]), biotechnical management (BTM; MD alone), chemical management (CM; three pyrethroid applications), and an untreated control (UC). MD targeting *S. nonagrioides* reduced egg mass densities by 37%–73% relative to UC across both years. Inundative *T. evanescens* releases at 10,000 parasitoids per decare (100,000 per hectare) achieved 92%–96% parasitism of *O. nubilalis* egg masses—providing larval suppression of this pest comparable to that achieved by chemical control—while leaving natural enemy activity intact. CM produced the highest grain yields (717–751 kg/da) but markedly suppressed natural parasitism of both borer species. IM yields (568–649 kg/da) exceeded UC (338–440 kg/da) by 40%–68% and were similar to BTM (552–622 kg/da). These findings demonstrate that combining MD with augmentative *T. evanescens* releases provides effective, pesticide-free management of both corn borer species simultaneously, offering a viable IPM alternative for Mediterranean maize production.

## Introduction

Maize (*Zea mays* L. (Poales: Poaceae)) is the most extensively produced cereal crop worldwide, with annual grain production exceeding 1.2 billion metric tons (FAO 2024). Maize, wheat, rice, and sugarcane together account for approximately half of total global primary crop production, underscoring the strategic importance of protecting maize yields at every stage of the production chain (FAO 2024). In Turkey, maize ranks second in cereal production following wheat, and production has increased substantially over the past decades, reaching ∼8.5 million tons in 2024. The Çukurova region of southern Turkey contributes roughly 20% of national maize production (TÜİK 2025).

Maize production is significantly constrained by insect pests, particularly corn borers, the European corn borer (ECB), *Ostrinia nubilalis* Hübner (Lepidoptera: Crambidae), and the Mediterranean corn borer (MCB), *Sesamia nonagrioides* Lefèbvre (Lepidoptera: Noctuidae), which are among the most destructive pests in Mediterranean maize-growing regions. *Ostrinia nubilalis* and *S. nonagrioides* are responsible for severe economic losses due to larval feeding within maize stalks and ears, with yield losses from corn borers reported at up to 30% under severe infestation pressure (Malvar et al. 2004, Meissle et al. 2010). Corn borer larvae exhibit cryptic behavior by boring into maize tissues until pupation, causing extensive tunneling damage that leads to both quantitative and qualitative yield losses (Battistelli et al. 2025). In addition to reducing grain yield, larval feeding facilitates the invasion of fungal pathogens, particularly *Fusarium* spp. and *Aspergillus* spp., which are associated with the accumulation of harmful mycotoxins, including fumonisins and deoxynivalenol, in maize kernels (Folcher et al. 2009, Bryła et al. 2022). The co-occurrence of *O. nubilalis* and *S. nonagrioides* on the same plants further exacerbates this risk, as both species act as vectors facilitating *Fusarium* entry into the ear (Arias-Martín et al. 2021). Globally, insect herbivores are estimated to cause ∼16% of yield losses in maize production (Oerke 2006), and these losses are projected to increase by 10%–25% per degree of mean surface warming as rising temperatures accelerate insect metabolic and population growth rates (Deutsch et al. 2018). Therefore, effective management of corn borers remains essential for sustainable maize production and food safety.

Several important natural enemies have been recorded for both species in the Çukurova region. The egg parasitoid *Trichogramma evanescens* Westwood (Hymenoptera: Trichogrammatidae) is the most effective natural enemy of *O. nubilalis* in the region, with natural parasitism rates occasionally reaching 60%, although this drops to ∼1% in fields with intensive insecticide use (Kayapınar and Kornoşor 1992). *Trichogramma* spp. are among the most widely mass-produced and deployed biological control agents globally, and their augmentative release against lepidopteran borers is well-established across multiple cropping systems (van Lenteren et al. 2018). Augmentative biological control encompasses both inoculative and inundative release strategies; in the present study, we use inundative release, involving a single mass release intended to directly overwhelm the target egg population, and this term is used consistently throughout to describe our treatment. Inundative releases of *T. evanescens* have been shown to reduce *O. nubilalis* infested plant numbers by up to 96% in Turkey (Öztemiz 2009). For *S. nonagrioides*, the egg parasitoid *Telenomus busseolae* (Gahan) (Hymenoptera: Scelionidae) is the dominant natural enemy across the Mediterranean basin, with natural parasitism rates of 24%–49% recorded in Çukurova, increasing toward the end of the growing season (Kornoşor et al. 1992, Kornoşor et al. 1995, Sertkaya, 1999). Sub-lethal exposure of *T. busseolae* to pyrethroid insecticides has been shown to significantly reduce its parasitism capacity and alter host-location behavior even at concentrations below the field-recommended dose (Bayram et al., 2010a, 2010b), highlighting the sensitivity of this key natural enemy to chemical inputs. Additional natural enemies of the stem borer complex are also present in the region, including the larval tachinid parasitoid *Lydella thompsoni*, several other hymenopteran larval and pupal parasitoids, and generalist egg predators such as anthocorid bugs (Meissle et al. 2010, Kaçar et al. 2023); although these were not the focus of the present study, they contribute to the broader natural enemy community whose conservation motivates the nonchemical treatments evaluated here.

The mating disruption (MD) technique disrupts pest reproduction by flooding the crop environment with synthetic sex pheromones, impeding males from locating females, reducing mating frequency, and thereby reducing subsequent egg-laying. This technique offers high selectivity for the target organism and does not adversely affect the activity of natural enemies (Witzgall et al. 2010). MD has gained increasing traction as a core component of integrated pest management (IPM) across a broad range of lepidopteran pests, particularly where regulatory constraints on synthetic insecticides are tightening (Kaçar et al. 2023). The sex pheromone of *S. nonagrioides* consists principally of (*Z*)-11-hexadecenyl acetate and (*Z*)-11-hexadecenal, with (*Z*)-11-hexadecen-1-ol and dodecyl acetate as minor components (Mazomenos, 1989; Sans et al., 1997). Field studies in Spain, France, and Greece have demonstrated that MD using the female pheromone of *S. nonagrioides* reduced male catches in pheromone monitoring traps by 60%–65% relative to untreated controls (UCs) and was at least as effective as insecticide application (Albajes et al. 2002). Large-scale field experiments using a ketone-based pheromone antagonist of *S. nonagrioides* further demonstrated 86%–90% reductions in the number of plants attacked during the second, most economically damaging generation (Solé et al. 2008). Pheromone antagonism between *S. nonagrioides* and *O. nubilalis* has been reported, with certain pheromone components of one species interfering with the chemical communication of the other (Eizaguirre et al. 2004, Gemeno et al. 2006), and this cross-species interference represents an important consideration for the design of any simultaneous dual-species MD program (Battistelli et al. 2025).

The combination of selective insecticides with augmentative biological control has been shown to outperform either method alone in some contexts (Gardner et al. 2011). However, synthetic pyrethroids commonly applied against corn borers in Turkey are known to be toxic to *T. evanescens* adults (Öztemiz 2001), which limits the compatibility of chemical and biological control approaches. A recent comprehensive review of management strategies for both corn borer species highlighted the growing interest in nonchemical alternatives, particularly in the context of the recent discovery of Bt resistance—which has revitalized interest in the biology and management of both pests—and increasing regulatory pressure on synthetic insecticides in Europe (Kaçar et al. 2023). Indeed, although no field resistance to Cry1Ab in *O. nubilalis* or *S. nonagrioides* had been detected through 2021, ongoing monitoring has been deemed essential as regional adoption of Bt maize in Spain has reached 64% in some areas (García et al. 2023). Against this backdrop, the development and validation of integrated, nonchemical management approaches at the regional level represents a research priority of both scientific and practical urgency.

Despite extensive research on each control method individually, comparative field trials integrating MD and augmentative releases of egg parasitoids against both stem borer species simultaneously are lacking in the Mediterranean region. Therefore, this study aimed to (i) compare the efficacy of integrated management (IM) (MD *+ T. evanescens* releases), biotechnical management (BTM) (MD alone), chemical management (CM) (insecticide applications), and an UC against *O. nubilalis* and *S. nonagrioides* in Çukurova; (ii) evaluate the compatibility of the component methods; and (iii) assess the implications of each approach for grain yield and parasitoid conservation.

## Materials and Methods

### Study Site and Experimental Design

Field experiments were conducted during the 2012 and 2013 second-crop maize (after wheat harvest) growing seasons in Mersin province, Çukurova region, southern Turkey (∼36°47′N, 34°36′E). Each year, one trial was established, and each trial consisted of two maize fields separated by a minimum distance of 1 km; the two trials were conducted on different fields, each was at least 30 decare (da; 1 da = 1,000 m^2^ = 0.1 ha), all within Mersin province. The hybrid cultivar P-41 (PR31P41; Pioneer Hi-Bred International), which is widely grown in Çukurova, was used in both years. The sowing dates were 24 June 2012 and 27 June 2013. Seeds were coated with imidacloprid (Gaucho FS 600; Bayer) and planted at 70 × 18 cm spacing using a precision seeder. This prophylactic neonicotinoid seed treatment, standard practice in the region at the time of the study, is now restricted in many jurisdictions because it offers limited yield benefit while posing a substantial risk to bees and other pollinators (Sgolastra et al. 2017); its use here reflects contemporary grower practice during the study years rather than a current recommendation.

The experimental area comprised two adjacent fields that were ∼1 km apart. UC and CM plots were established in one field, while BTM and IM plots were established in the other (Fig. 1). This spatial separation was essential for the validity of the MD treatment and to prevent *T. evanescens* dispersal between the treatments. A minimum 100 m buffer strip of maize was maintained between the BTM and IM plots to further limit parasitoid movement between them (Gardner et al. 2011). Within each treatment area, three sampling sub-plots were established as fixed sampling stations distributed across the treatment plot and separated from one another by at least 20 m, each kept in the same position for the duration of the season; each station defined a 2,000-m^2^ assessment area within which the weekly plant sampling and harvest measurements were carried out, so the three sub-plots measured within-plot spatial variability. Because MD requires large contiguous treated areas, interspersed replication of whole treatments was not feasible, a constraint inherent to large-plot MD research (Hurlbert 1984).

**Fig. 1.**
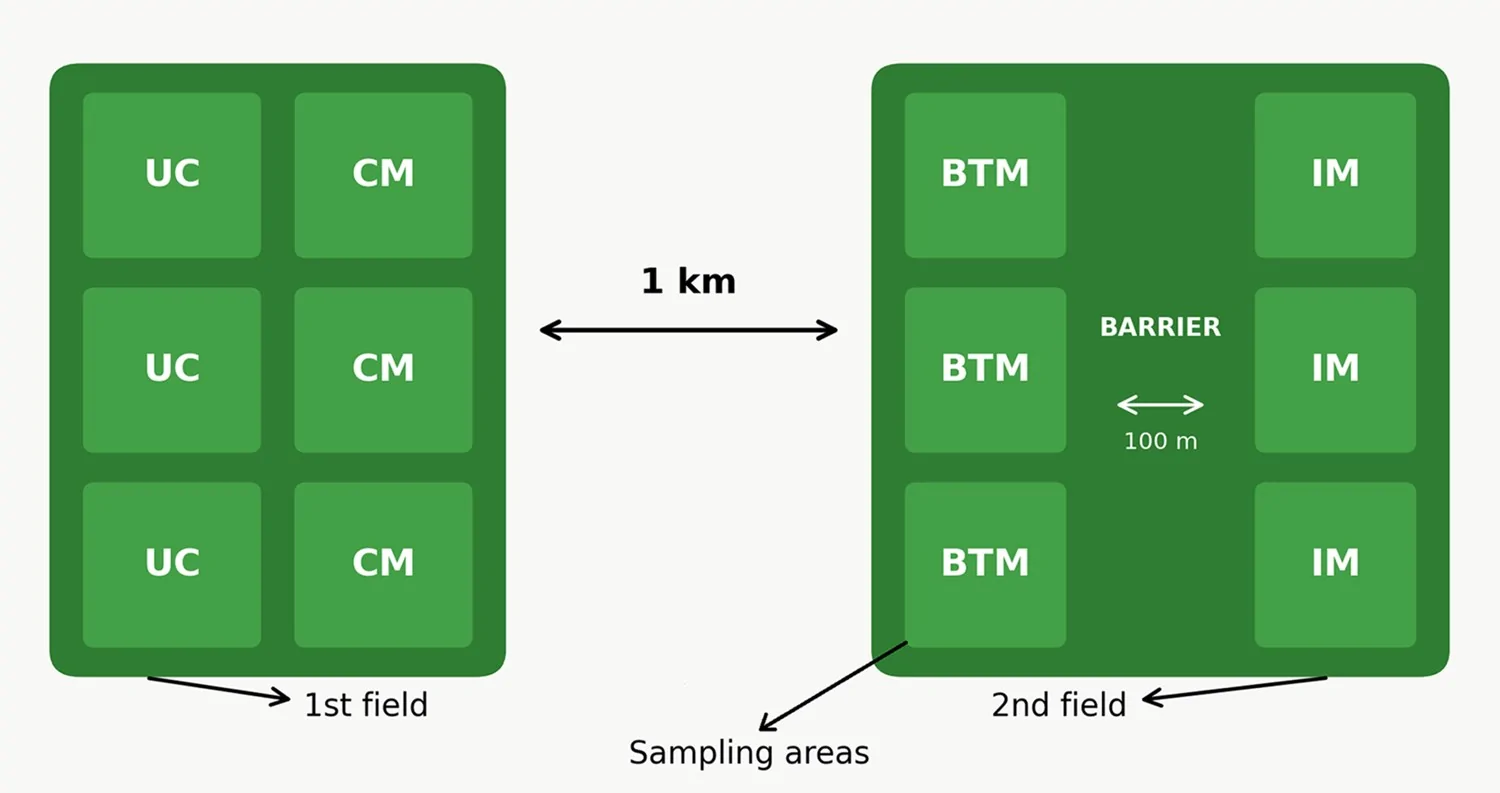
Experimental plot layout of the field trials. BTM: biotechnical management plots; IM: integrated management plots; CM: chemical management plots; UC: untreated control plots.

Standard agronomic practices were uniformly applied across all the plots. Basal fertilization consisted of 80 kg/da of 15:15:15 N:P:K at sowing, with 30 kg/da of 46% urea applied as top-dressing at the six-leaf stage. Furrow irrigation was applied every 8–10 days. In both years, an early season outbreak of beet armyworm, *Spodoptera exigua* (Hübner) (Lepidoptera: Noctuidae), was controlled with emamectin benzoate (Proclaim; Syngenta) at 40 g/da applied to all plots on 9 July 2012 and 6 July 2013, before stem borer populations reached economically significant densities. Because the imidacloprid seed treatment and this emamectin benzoate application were applied uniformly to every plot, the UC was untreated only with respect to corn borer management; any residual effects of these early-season inputs on the natural enemy community would therefore have been common to all four treatments.

## Treatment Descriptions

### Chemical Management

The CM plots received three insecticide applications per season, timed according to adult monitoring via light traps. In both 2012 and 2013, the first and third applications used lambda-cyhalothrin (Karate Zeon; Syngenta, 50 SC, 30 ml/da) and the second used deltamethrin (Decis; Bayer, 25 EC, 50 ml/da). Application dates were 6 August, 20 August, and 22 September in 2012, and 4 August, 20 August, and 15 September in 2013.

### Biotechnical Management

MD targeting *S. nonagrioides* was implemented using commercial polyvinyl chloride dispensers (SESATEC; SEDQ, Barcelona, Spain) loaded with the two pheromone components of this species: (*Z*)-11-hexadecenyl acetate and (*Z*)-11-hexadecen-1-ol (Krokos et al., 2002; Sans et al., 1997). Dispensers were attached to plants at ∼1.2 m height using plastic clips, at a density of 10 per da. Dispensers had a 90-day active life and remained in place until harvest. Installation dates were 13 August 2012 and 2 August 2013. Buffer strips between BTM and IM plots also received dispensers. No biological or chemical control measures against corn borers were applied in BTM and IM plots.

### Integrated Management

IM plots received both MD (as described above) and inundative releases of *T. evanescens* against *O. nubilalis*. The parasitoid was reared on *Ephestia kuehniella* Zeller (Lepidoptera: Pyralidae) eggs under controlled laboratory conditions (25 ± 1 °C, 65% ± 5% RH, 16:8 L:D). Species identity of the *Trichogramma* colony was confirmed as *T. evanescens* by Dr. Gennaro Viggiani, University of Naples Federico II. Releases were made using release sachets (supplied by the Adana Biological Control Research Institute), each containing ∼1,500 UV-sterilized *E. kuehniella* eggs parasitized by *T. evanescens*. A single inundative release of 10,000 parasitoids per decare (100,000 parasitoids per hectare; ∼20 sachets per plot) was made in each year, timed to coincide with the first detection of *O. nubilalis* egg masses on plants following light trap monitoring. Release dates were 6 August in both 2012 and 2013.

### Pest Monitoring

Adult populations of both corn borer species were monitored throughout the season using pheromone bucket traps (Sesalab and Ostrilab E/Z; SEDQ, Barcelona, Spain) placed in each plot at 20 m intervals, and a Robinson-type light trap positioned at the field margin. Weekly trap catches were recorded and adults removed. Species- and sex-specific pheromone traps and the nonspecific light trap were used in combination because each method has complementary limitations: pheromone traps attract only responsive males of the target species/race and can lose sensitivity under MD, whereas light traps sample both sexes of multiple species nonselectively and were retained here as an independent check on adult activity, particularly for *O. nubilalis* where pheromone trap catches proved uninformative (see Effects on Adult Populations). Since *O. nubilalis* exists as two pheromone races (Z race and E race; Klun et al. 1973, Klun and Maini, 1979), traps baited with both race-specific pheromones were used; however, only Z-race traps yielded positive catches in the study area, consistent with our unpublished survey data.

Egg mass counts for both species commenced upon first detection of adults in light traps. Sampling was conducted weekly by examining 100 plants per plot (five plants at each of 20 randomly selected points). All leaf surfaces were inspected for *O. nubilalis* egg masses, while leaf sheaths and the inner surfaces of husks were examined for *S. nonagrioides* egg masses. Parasitism rates of *O. nubilalis* egg masses were determined ∼1 wk after each *T. evanescens* release. Parasitism of *S. nonagrioides* egg masses was assessed four times per season (twice per generation) during peak egg-laying periods. Egg masses were scored as parasitized when eggs exhibited blackening consistent with parasitoid development. Freshly collected egg masses were transported to the laboratory in Falcon tubes and monitored for blackening.

### Plant Damage Assessment

Larval populations within plants were assessed at harvest. At harvest, a minimum of 120 plants per plot were cut at soil level, transported to the laboratory, and assessed for: plant height (measured from first internode to tassel tip), stem diameter at the first internode (measured with digital calipers), number of borer entry holes per plant, total gallery length (measured by longitudinal dissection of the stem), *S. nonagrioides* and *O. nubilalis* larval counts per plant, and proportion of ears with feeding damage (infested ears), including plants from which ears had fallen as a result of larval tunneling of the peduncle.

### Yield and Quality Assessment

At harvest, 120 ears were collected from each plot and threshed. Grain weight was recorded and yield extrapolated to kg per da. Sub-samples of ∼100 g per plot were milled and submitted for protein content analysis by the Food Science Department, Çukurova University in 2012 and the East Mediterranean Agricultural Research Institute in 2013.

### Statistical Analysis

Each treatment occupied a single large, contiguous plot per year, as required to sustain an effective mating-disruption atmosphere; whole-plot replication is therefore not feasible, and the three sub-plots within each plot are nested pseudoreplicates rather than independent replicates of the treatment (Hurlbert 1984). Treatments were also only partly interspersed between the two fields (UC and CM in one, BTM and IM in the other), so treatment is partly confounded with field. We therefore treat all tests as exploratory, basing inference on effect sizes and on the consistency of treatment effects across years rather than on formal field-scale replication. Because the 2012 and 2013 trials were established on different fields, analyzing each year separately cannot distinguish a treatment effect from an idiosyncrasy of that year’s field or weather; we therefore followed a joint analysis, pooling generations to a seasonal value per sub-plot and modeling each response across both years simultaneously, with application, year, and their interaction as fixed effects. This lets year-to-year consistency (or its absence) be tested formally rather than only described narratively, and partly disentangles treatment from field-position and environmental effects, as recommended for this design. Error structures were chosen per response: quasi-Poisson for egg-mass counts (dispersion estimated from the Pearson χ^2^, since the negative-binomial dispersion parameter could not be estimated reliably from only three sub-plots per treatment-by-year cell), quasi-binomial for parasitism and ear-infestation proportions, and Gaussian, on ln(*x* + 1)- or arcsine-√-transformed data as appropriate, for the remaining continuous measurements (Zar 2010). For each response, we first tested the application × year interaction: where it was not significant, treatment means were pooled across years and separated by Tukey’s HSD (reported as “combined-year” separations); where it was significant, indicating that the treatment ranking differed between years, we retained within-year simple-effect comparisons, as in the original single-year analyses. Even where the joint test was significant, sub-plots remain nested pseudoreplicates within a single plot per treatment per year, so a significant result shows that a pattern held across the two independently sited trials, not that it has been demonstrated with true field-scale replication; conclusions are still offered with this caveat. Under this joint framework, the seasonal *S. nonagrioides* egg-mass reduction (Table 1) showed a significant application × year interaction (quasi-Poisson *F *= 22.8; df = 3, 16, *P *< 0.001), consistent with the larger suppression achieved in 2012 than in 2013 described below, rather than being read only as a large but formally nonsignificant effect size. Efficacy against *S. nonagrioides* is the percentage reduction relative to UC. Descriptive statistics and the original single-year comparisons used R 4.3.2; the joint quasi-Poisson, quasi-binomial, and Gaussian models added were fitted in Python.

**Table 1.** Mean *Sesamia nonagrioides* egg-mass density (EP/100 plants; seasonal totals across both generations, mean ± SE), with efficacy relative to the UC in parentheses, 2012–2013

	Treatment			
Year	IM	BTM	CM	UC
**2012**	61.7 ± 4.7 (73.0) *b*	71.0 ± 6.8 (68.9) *b*	62.0 ± 4.2 (72.8) *b*	228.0 ± 19.5 *a*
**2013**	127.7 ± 6.4 (36.5) *b*	124.7 ± 5.9 (38.0) *b*	146.3 ± 9.9 (27.2) *b*	201.0 ± 14.0 *a*

Values are seasonal egg-mass density (EP/100 plants; summed across all sampling dates of both generations), mean ± SE across the three sub-plots, with the percentage reduction relative to UC (seasonal efficacy) in parentheses. Means not sharing a letter within a year differed in an exploratory Tukey HSD test (*P *< 0.05); η^2^ = 0.96 (2012) and 0.84 (2013). A joint quasi-Poisson model across both years found a significant application × year interaction (*F *= 22.8; df = 3, 16, *P *< 0.001), so letters above are within-year simple-effect separations rather than a pooled comparison. IM, integrated management; BTM, biotechnical management; CM, chemical management; UC, untreated control.

## Results

### Effects on *S. nonagrioides* Egg Populations

In both years, *S. nonagrioides* completed two distinct egg-laying periods within the sampling window, corresponding to the third and fourth generations of the pest, consistent with earlier phenological records from the region (Sertkaya 1993). In 2012, egg populations in UC plots reached a peak weekly density of 41.7 egg masses (EP)/100 plants in the third generation and 87.0 EP/100 plants in the fourth generation; seasonal totals for all treatments are reported in Table 1. All treatments reduced egg mass counts relative to the UC (Table 1). Total seasonal efficacy against *S. nonagrioides* egg populations was 73.0%, 68.9%, and 72.8% for IM, BTM, and CM plots, respectively; the three treatments were similar to one another and all carried far fewer egg masses than UC (η^2^ = 0.96). In 2013, UC peak egg populations were lower (29.0 EP/100 plants in generation 3; 80.3 EP/100 plants in generation 4). Mating-disruption plots again carried fewer egg masses than UC (IM: 36.5%; BTM: 38.0% seasonal efficacy), while CM plots showed 27.2% efficacy (η^2^ = 0.84); as in 2012, the treatments were similar to one another. The lower efficacy of MD in 2013 compared with 2012 may reflect higher initial pest pressure and possible immigration of mated females from adjacent silage maize fields, consistent with the known sensitivity of MD to initial population density (Cardé and Minks 1995). Modeling both years jointly confirmed a significant application × year interaction for seasonal egg-mass counts (quasi-Poisson *F *= 22.8; df = 3, 16, *P *< 0.001), i.e. the magnitude of egg-mass suppression by all three managed treatments genuinely differed between years rather than reflecting sampling noise around a constant effect, reinforcing the interpretation above.

Insecticides applied in CM plots are not expected to directly reduce adult populations or egg-laying behavior; the observed lower egg counts in CM versus UC plots may reflect a behavioral preference of egg-laying females for the adjacent UC plots, a possible edge effect attributable to the physical proximity of these two treatments within the same field. This interpretation is offered as a hypothesis only: oviposition preference and female movement were not directly measured in this study, and the relative spatial position of the CM and UC plots was not manipulated. It should also be noted that the apparent CM reduction relative to UC was much smaller in 2013 (27.2% seasonal efficacy) than in 2012 (72.8%), and given the absence of treatment-level replication, the smaller 2013 value cannot be confidently distinguished from sampling variation.

### Effects on *O. nubilalis* Egg Populations

*Ostrinia nubilalis* completed one egg-laying period in 2012 and two in 2013. Egg mass densities across all treatments were low compared with *S. nonagrioides*. No significant differences in *O. nubilalis* egg mass counts were detected among treatments in either year (Table 2). The *S. nonagrioides* MD dispensers used in this study contained (*Z*)-11-hexadecenyl acetate and (*Z*)-11-hexadecen-1-ol but did not include (*Z*)-11-hexadecenal, the component responsible for *O. nubilalis* pheromone antagonism (Gemeno et al. 2006). Accordingly, no cross-species MD effect on *O. nubilalis* was expected or observed. Unlike the other response variables, only treatment-level weekly means and standard errors were retained for this variable, not the underlying per-sub-plot counts, so a joint application × year model of the kind used elsewhere could not be fitted for *O. nubilalis* egg counts; we recommend that the sub-plot-level counts be archived alongside future datasets so this comparison can be completed.

**Table 2.** Egg mass counts of *Ostrinia nubilalis* (EP/100 plants ± SE) by treatment and year

Year	IM	BTM	CM	UC
**2012**	7.3 ± 0.9 *a*	7.7 ± 0.9 *a*	6.3 ± 1.0 *a*	8.3 ± 1.5 *a*
**2013**	10.3 ± 1.0 *a*	10.3 ± 1.1 *a*	10.0 ± 1.0 *a*	11.3 ± 1.5 *a*

In exploratory one-way ANOVA, treatment means did not differ within either year (means share the letter a; *P* > 0.05); these comparisons are exploratory only; the three sub-plots are nested pseudoreplicates, not independent replicates (see Statistical Analysis). Means ± SE pooled across sampling dates. A joint two-year model could not be fitted for this variable because only treatment-level weekly means and SEs, not sub-plot-level counts, were retained in the archived dataset. IM, integrated management; BTM, biotechnical management; CM, chemical management; UC, untreated control.

### Effects on Adult Populations

Pheromone trap catches of *S. nonagrioides* in IM and BTM plots were consistently and substantially lower than in UC and CM plots across both years. In 2012, peak catches in UC and CM plots were 177.3 and 118.7 adults/trap/wk, respectively, while IM and BTM plots recorded maxima of only 2.7 and 4.3 adults/trap/wk. In 2013, *S. nonagrioides* catches were negligible across all plots, and monitoring traps in IM and BTM plots captured no adults. This finding is consistent with published information that pheromone monitoring traps lose sensitivity in the presence of MD dispensers, as the high-density point sources of pheromone outcompete the lower-output rubber septum monitoring lures (Laurent and Frérot 2007). Accordingly, adult pheromone trap data from MD plots should not be used to estimate population density, and alternative monitoring tools (e.g. light traps) are recommended; while these may not accurately reflect absolute density, they successfully capture population peaks and fluctuations.

*O. nubilalis* adults were captured in neither Race E nor Race Z pheromone traps in any plot across both years, confirming that light traps are a more reliable monitoring tool for this species in Çukurova. The bucket-style pheromone traps used here may partly explain this poor performance, since the small-funnel trap designs commonly used for corn borer monitoring are generally less efficient than the larger, purpose-built Heliothis-style traps; future monitoring efforts in this system would benefit from re-evaluating trap design (Maini and Burgio 1991, Burgio and Maini 1994).

### Parasitism of Egg Masses

Parasitism of *S. nonagrioides* egg masses by *Telenomus busseolae* was high in all nonchemical treatments and lowest where insecticides were applied (Table 3). Pooled across generations, seasonal parasitism in 2012 was similar in the integrated (55.0%), biotechnical (52.6%), and untreated (47.2%) plots and much lower in the chemical plots (25.2%); the same pattern held in 2013 (IM 21.3%, BTM 22.0%, UC 21.5%, CM 11.4%). Parasitism differed among applications (*P *< 0.001) and between years (*P *< 0.001), with a significant application × year interaction (*P *= 0.018); however, integrated and BTM did not differ from the UC, and only CM was significantly lower. This suppression in CM plots is consistent with the known deleterious effects of synthetic pyrethroids on *T. busseolae* adults (Bayram et al. 2010a).

**Table 3.** Mean parasitism (%) of *Sesamia nonagrioides* egg masses by *Telenomus busseolae* by treatment and year (generations pooled to seasonal values)

Year	IM	BTM	CM	UC
**2012**	55.0 ± 3.0 *a*	52.6 ± 2.2 *a*	25.2 ± 4.1 *b*	47.2 ± 2.0 *a*
**2013**	21.3 ± 0.3 *a*	22.0 ± 1.7 *a*	11.4 ± 1.5 *b*	21.5 ± 0.8 *a*

Seasonal parasitism (%), generations pooled; values are arithmetic means ± SE on the percentage scale, while ANOVA, Tukey HSD, and η^2^ were computed on arcsine √-transformed proportions. Integrated and BTM did not differ from the UC; only CM was lower. Means not sharing a letter within a year differed in an exploratory Tukey HSD test (*P *< 0.05); η^2^ = 0.88 (2012) and 0.87 (2013). In the pooled two-way model, application *P *< 0.001, year *P *< 0.001, and application × year *P *= 0.018. This variable was already analyzed with a joint application × year model in the original submission; the same joint approach has now been extended to Tables 1, 2, 4, and 5. IM, integrated management; BTM, biotechnical management; CM, chemical management; UC, untreated control.

Parasitism of *O. nubilalis* egg masses by *T. evanescens* differed markedly among treatments in both years, with treatment accounting for the large majority of the variation (descriptive η^2^ = 0.90 in both years; Table 4). In IM plots, parasitism reached 95.8% in 2012 and 92.1% in 2013—far exceeding BTM (21.4%, 26.4%), CM (13.3%, 15.6%), and UC (18.1%, 30.1%). Chemical applications in CM plots suppressed *T. evanescens* activity, consistent with established toxicity of lambda-cyhalothrin and deltamethrin to this parasitoid (Öztemiz 2001). The high natural parasitism observed in UC plots in 2013 is likely attributable to the diverse lepidopteran host complex available in the surrounding landscape (greenhouse-grown Spodoptera species on open-field vegetables), which supports elevated resident *T. evanescens* populations in this area. A joint quasi-binomial model fitted to the sub-plot-level egg counts across both years found no significant application × year interaction (*F *= 0.40; df = 3, 16, *P *= 0.75), confirming that the very large IM advantage was consistent between years rather than driven by either year alone (pooled application effect: *F *= 50.4; df = 3, 19, *P *< 0.001; year effect not significant, *F *= 0.86; df = 1, 19, *P *= 0.37).

**Table 4.** Mean parasitism (%) of *Ostrinia nubilalis* egg masses by *Trichogramma evanescens* releases in IM and naturally in other treatments

Year	IM	BTM	CM	UC
**2012**	95.8 ± 4.2 *a*	21.4 ± 6.0 *b*	13.3 ± 6.7 *b*	18.1 ± 1.9 *b*
**2013**	92.1 ± 4.0 *a*	26.4 ± 6.1 *b*	15.6 ± 2.3 *b*	30.1 ± 10.0 *b*

Values are arithmetic means ± SE on the percentage scale; ANOVA, Tukey HSD, and η^2^ were computed on arcsine √-transformed proportions. IM (inundative *Trichogramma evanescens* releases) greatly exceeded all other treatments, which did not differ from one another (η^2^ = 0.90 in both years; binomial GLM application *P *< 0.001). Means not sharing a letter within a year differed in an exploratory Tukey HSD test (*P *< 0.05). A joint quasi-binomial model fitted to both years together found no significant application × year interaction (*F *= 0.40; df = 3, 16, *P *= 0.75), so the IM advantage is treated as a single, pooled effect across years (application effect *F *= 50.4; df = 3, 19, *P *< 0.001; year *P *= 0.37). IM, integrated management; BTM, biotechnical management; CM, chemical management; UC, untreated control.

### Plant Damage at Harvest

Plants in managed plots were consistently taller than those in UC plots, a difference we attribute to reduced stem borer injury rather than differential crop growth (Table 5). In 2012, mean plant height was significantly greater in IM (228.1 cm), BTM (223.3 cm), and CM (221.9 cm) plots compared with UC plots (204.6 cm; *P *= 0.004). In 2013, numerical differences in the same direction were observed but did not reach statistical significance. A joint model across both years showed no significant application × year interaction for plant height (*F *= 0.20; df = 3, 16, *P *= 0.89), so the 2013 nonsignificance most likely reflects reduced power in a single year rather than a genuinely different treatment effect: pooling both years, UC plants (206.6 cm) were significantly shorter than all three managed treatments (222.3–226.8 cm; combined-year Tukey, *P *< 0.02 for each comparison with UC), which did not differ from one another (application effect *F *= 6.34; df = 3, 16, *P *= 0.005).

**Table 5.** Plant and ear damage parameters at harvest by treatment, 2012–2013

Parameter	Year	IM	BTM	CM	UC
**Plant height (cm)**	2012	228.1 ± 4.2 *a*	223.3 ± 3.8 *a*	221.9 ± 2.1 *a*	204.6 ± 2.4 *b*
	2013	225.5 ± 3.8 *a*	221.3 ± 7.4 *a*	223.6 ± 4.6 *a*	208.7 ± 8.2 *a*
**Stem diameter (cm)**	2012	2.2 ± 0.0 *ab*	2.1 ± 0.1 *ab*	2.3 ± 0.0 *a*	2.0 ± 0.0 *b*
	2013	2.1 ± 0.0 *a*	2.1 ± 0.0 *a*	2.2 ± 0.0 *a*	2.1 ± 0.0 *a*
**Entry holes/plant**	2012	15.2 ± 0.5 *b*	16.2 ± 0.5 *ab*	12.5 ± 0.9 *c*	19.0 ± 0.6 *a*
	2013	15.7 ± 0.7 *a*	16.6 ± 0.3 *a*	15.3 ± 0.7 *a*	18.0 ± 0.7 *a*
**Gallery length (cm)**	2012	82.5 ± 2.6 *ab*	86.1 ± 0.9 *ab*	76.2 ± 3.9 *b*	93.5 ± 1.2 *a*
	2013	81.5 ± 2.1 *ab*	85.4 ± 1.6 *ab*	77.0 ± 2.5 *b*	89.2 ± 2.5 *a*
***Sesamia nonagrioides* larvae/plant**	2012	2.4 ± 0.0 *a*	2.4 ± 0.2 *a*	2.1 ± 0.1 *a*	2.6 ± 0.1 *a*
	2013	2.3 ± 0.1 *a*	2.4 ± 0.0 *a*	2.3 ± 0.1 *a*	2.5 ± 0.0 *a*
***Ostrinia nubilalis* larvae/plant**	2012	0.1 ± 0.0 *b*	0.4 ± 0.0 *a*	0.1 ± 0.0 *b*	0.5 ± 0.0 *a*
	2013	1.0 ± 0.1 *a*	1.1 ± 0.1 *a*	1.1 ± 0.0 *a*	1.1 ± 0.1 *a*
**Ear infestation (proportion)**	2012	0.5 ± 0.0 *b*	0.5 ± 0.0 *b*	0.3 ± 0.0 *c*	0.8 ± 0.0 *a*
	2013	0.5 ± 0.0 *b*	0.6 ± 0.0 *b*	0.4 ± 0.0 *c*	0.8 ± 0.0 *a*

Means ± SE. Separation indices (η^2^), 2012 and 2013: plant height 0.79, 0.36; stem diameter 0.69, 0.49; entry holes 0.86, 0.56; gallery length 0.76, 0.68; *S. nonagrioides* larvae 0.43, 0.50; *O. nubilalis* larvae 0.98, 0.05; ear infestation 0.95, 0.94. Proportions and counts were transformed before analysis. Means not sharing a letter within a row differed in an exploratory Tukey HSD test (*P *< 0.05). Joint application × year models found no significant interaction for plant height (*P *= 0.89), stem diameter (*P *= 0.30), gallery length (*P *= 0.75), *S. nonagrioides* larvae (*P *= 0.61), or ear infestation (*P *= 0.67)—for these variables the row-by-row separations above are consistent with a single, pooled treatment ranking across years. Entry holes/plant (*P *= 0.042) and *O. nubilalis* larvae/plant (*P *< 0.001) showed a significant interaction, confirming that the treatment differences visible in 2012 for these two variables genuinely did not persist in 2013. IM, integrated management; BTM, biotechnical management; CM, chemical management; UC, untreated control.

Gallery length per plant was significantly shorter in CM plots (76.2 cm in 2012; 77.0 cm in 2013) compared with UC plots (93.5 and 89.2 cm), while IM and BTM plots were intermediate. *S. nonagrioides* larval densities at harvest did not differ significantly among treatments in either year (Table 5), reflecting the characteristic overwintering behavior of this species, in which a fraction of the population ceases feeding and overwinters in the stem regardless of earlier treatment effects on egg-laying. Overwintering survival in this stage is variable and can be substantial under severe winters, and some authors distinguish this photoperiod-induced arrest from the deeper, more cold-hardy diapause reported for a fraction of the *O. nubilalis* population; this distinction does not affect our interpretation of the harvest counts. *O. nubilalis* larval counts at harvest were significantly lower in IM (0.07 larva/plant) and CM (0.05 larva/plant) plots than in BTM (0.37) and UC (0.45) plots in 2012, confirming the efficacy of both *T. evanescens* releases and insecticide applications against this pest. In 2013, when *O. nubilalis* completed an additional generation, larval densities increased across all plots (1.03–1.08 larva/plant), and no significant differences among treatments were detected. Joint two-year models supported these year-specific readings for ­gallery length and for *S. nonagrioides* larvae, both of which showed no application × year interaction (gallery length: *F *= 0.41; df = 3, 16, *P *= 0.75, application effect *P *< 0.001; *S. nonagrioides* larvae: *F *= 0.62; df = 3, 16, *P *= 0.61, application effect *P *= 0.032, driven only by a CM–UC difference). By contrast, *O. nubilalis* larvae and entry holes/plant both showed a significant application × year interaction (df = 3, 16 for both; *P *< 0.001 for *O. nubilalis* larvae; *P *= 0.042 for entry holes), confirming statistically that the treatment differences visible in 2012 for these two variables were genuinely absent in 2013 rather than merely underpowered.

Ear infestation rates revealed the most consistent treatment effects across years (Table 5). In 2012, infested ear proportions were 0.5, 0.5, 0.3, and 0.8 in IM, BTM, CM, and UC plots, respectively. In 2013, values were 0.5, 0.6, 0.4, and 0.8. In both years, CM plots showed significantly lower ear infestation than IM and BTM plots, which in turn showed significantly lower infestation than UC. IM and BTM plots did not differ significantly from one another, confirming that the MD pheromone dispensers—rather than *T. evanescens* releases—were primarily responsible for the reduction in ear infestation, given that *T. evanescens* targets *O. nubilalis* eggs but does not suppress *S. nonagrioides* oviposition or larval access to the ear. A joint model confirmed this pattern was consistent across years, with no significant application × year interaction (*F *= 0.53; df = 3, 16, *P *= 0.67) and a highly significant, consistent application effect (*F *= 84.8; df = 3, 16, *P *< 0.001), the strongest of any response variable examined in this study.

### Grain Yield and Quality

Grain yield and protein content separated clearly among treatments in both years, with treatment accounting for most of the variation (descriptive η^2^ for yield = 0.86 in 2012 and 0.94 in 2013; Fig. 2). In 2012, yield was highest in CM plots (750.6 kg/da), followed by IM (648.6 kg/da), BTM (621.8 kg/da), and UC (440.4 kg/da). CM plots yielded ∼70% more than UC plots; IM and BTM plots yielded 47% and 41% more than UC, respectively. In 2013, the same rank order was maintained: CM (716.9 kg/da) > IM (568.4 kg/da) > BTM (552.1 kg/da) > UC (337.7 kg/da), with yield advantages of 112%, 69%, and 64% over UC for CM, IM, and BTM, respectively. A joint model across both years found no significant application × year interaction for yield (*F *= 0.51; df = 3, 16, *P *= 0.68), so the CM > IM ≈ BTM > UC ranking held consistently in both years and the pooled application effect is correspondingly strong (*F *= 50.3; df = 3, 16, *P *< 0.001), with a significant but comparatively minor year effect (*P *= 0.003) reflecting the somewhat lower yields recorded in 2013 across all four treatments.

**Fig. 2.**
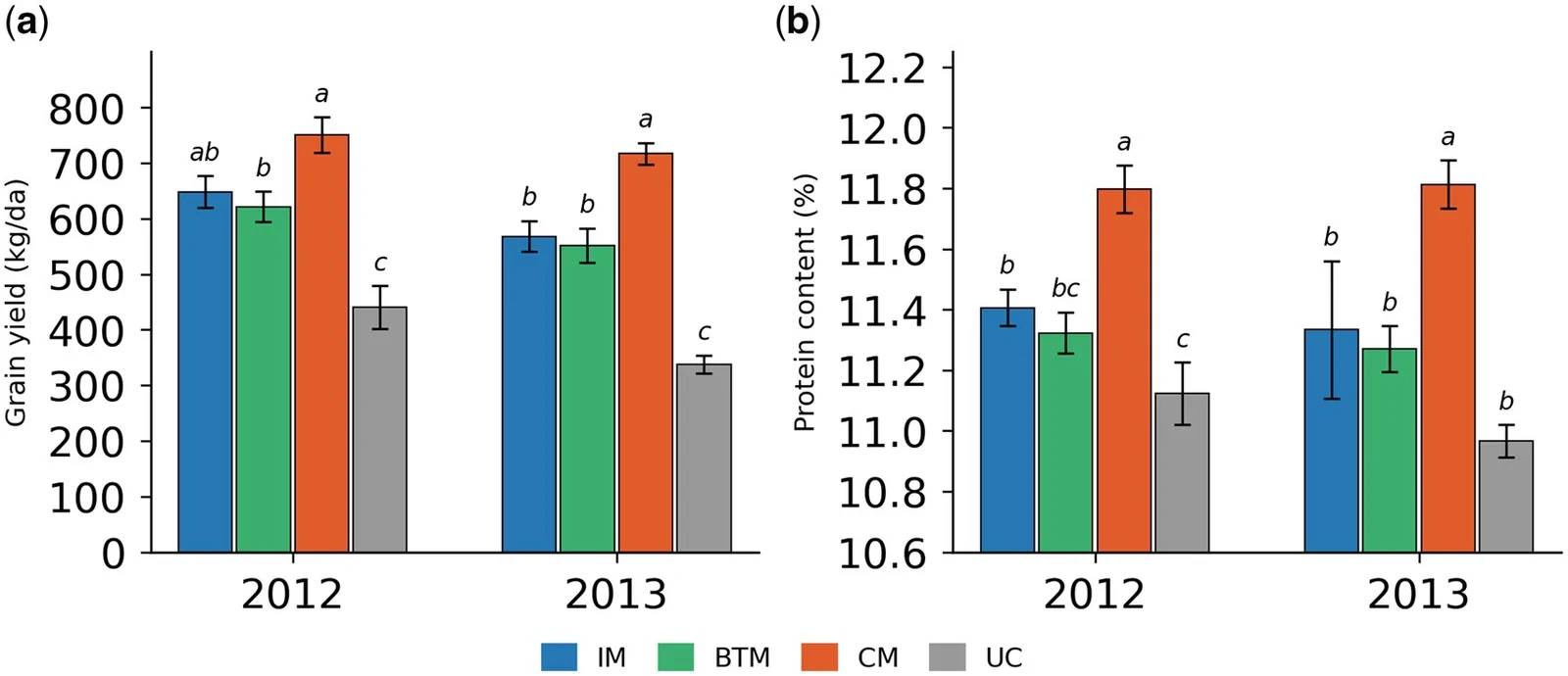
(A) Grain yield (kg/da) and (B) protein content (%) of maize grain by treatment and year (mean + SE, *n* = 3 sub-plots). The separation among treatment means was large (descriptive separation index η^2^: yield 0.86 in 2012, 0.94 in 2013; protein 0.83 and 0.74). Joint application × year models found no significant interaction for either yield (*P *= 0.68) or protein (*P *= 0.88), so a single, pooled ranking (CM > IM ≈ BTM > UC) applies consistently across both years (application effect *P* < 0.001 for both). IM, integrated management; BTM, biotechnical management; CM, chemical management; UC, untreated control.

Grain protein content followed a similar pattern, with CM plots producing significantly higher protein content than UC plots in both years (2012: 11.8% vs. 11.1%; 2013: 11.8% vs. 11.0%). IM and BTM plots did not differ significantly from each other in protein content in either year. The absence of a significant yield or protein difference between IM and BTM plots is consistent with the finding that *T. evanescens* releases effectively suppressed *O. nubilalis* larval damage, but neither MD nor biological control adequately suppressed the heavier yield impact of *S. nonagrioides*, which was the dominant pest across the season. Grain protein followed the same pattern of year-consistency: the application × year interaction was not significant (*F *= 0.22; df = 3, 16, *P *= 0.88), supporting a single, pooled CM > IM ≈ BTM > UC ranking across both years (application effect *F *= 17.5; df = 3, 16, *P *< 0.001).

Overall grain yields were low in all treatments across both years, attributable primarily to the intensity of MCB infestation in this second-crop production system.

## Discussion

This two-year study demonstrates that MD with *S. nonagrioides* pheromone dispensers and inundative releases of *T. evanescens* can be combined as an effective, insecticide-free strategy for managing both principal corn borer species in second-crop maize in the Çukurova region. The two methods were fully compatible, mutually reinforcing, and individually targeted at separate pest species.

MD reduced *S. nonagrioides* egg mass numbers by 37%–73% relative to UC across both years, a range consistent with earlier European field studies using the same pheromone components. Albajes et al. (2002) reported 60%–65% reductions in male captures in Spain, France, and Greece using sprayable pheromone formulations. The solid dispensers used in the present study showed comparable or superior performance in 2012 (68%–73% efficacy) and somewhat lower efficacy in 2013 (37%–38%). Year-to-year variability in MD efficacy is well documented (Cardé and Minks 1995) and is influenced by initial pest density, immigration of mated females from untreated areas, dispenser temperature sensitivity, and environmental conditions during the season. The immigration of already-mated females from adjacent untreated fields is widely recognized as one of the primary causes of MD failure or reduced efficacy, regardless of pheromone formulation quality, and its influence can dominate within-season variability in outcomes (Witzgall et al. 2010). In both years studied here, silage maize fields adjacent to the treatment plots likely served as immigration sources for mated females, as silage maize growers typically cease insecticide application during grain fill—a period that coincides with peak *S. nonagrioides* oviposition. The lower efficacy in 2013 may also reflect less favorable weather during the MD pheromone dispenser application period. Mean daily temperatures and wind speeds were somewhat higher than in 2012, conditions that accelerate the release and atmospheric dispersal of pheromone from dispensers.

The absence of a detectable cross-species MD effect on *O. nubilalis* in the present study is consistent with the pheromone chemistry of the dispensers used. Gemeno et al. (2006) demonstrated that the *O. nubilalis* pheromone antagonism attributed to *S. nonagrioides* pheromone is mediated specifically by the (*Z*)-11-hexadecenal component, which was not present in the SESATEC dispensers used here. Future studies could explore whether inclusion of this component enhances the dual-species efficacy of the MD treatment.

*T. evanescens* releases at 10,000 parasitoids per decare (100,000 per hectare) achieved remarkably *consistent O. nubilalis* egg mass parasitism rates of 92%–96% across both years, comparable to rates reported in earlier Çukurova trials using similar release doses (Öztemiz 2009). By comparison, natural parasitism without augmentative releases (i.e. in BTM, CM, and UC plots, which received no *T. evanescens* releases) ranged from only 13%–30% across years (Table 4), underscoring the added parasitism achieved through inundative release. The high consistency of parasitism rates across both seasons is noteworthy and aligns with the broader literature showing that inundative *Trichogramma* releases in maize can achieve stable, high-level egg parasitism when release timing is well-matched to host egg-laying phenology (van Lenteren et al. 2018). CM plots achieved equivalent suppression of *O. nubilalis* larval populations in 2012, confirming that three well-timed pyrethroid applications represent effective CM for this pest. However, insecticide use depressed natural egg parasitism: parasitism of *S. nonagrioides* by *T. busseolae* was significantly lower in CM than in the untreated and mating-disruption plots (Table 3), and natural parasitism of *O. nubilalis* by *T. evanescens* was also lowest in CM, although the nonrelease plots did not differ significantly (Table 4). This finding underscores a fundamental limitation of chemical control in this system: while effective against target pests in the short term, pyrethroid applications disrupt the natural enemy community that would otherwise provide partial suppression of pest populations throughout the season, and probably carry other negative side effects, such as harm to bees and other pollinators; consequently, insecticide applications during maize blooming are now restricted in several countries. The suppression of arthropod natural enemies by pyrethroid applications in maize is well established and extends beyond egg parasitoids to include predators and larval parasitoids, with the natural enemy community in pyrethroid-sprayed fields consistently showing lower abundance and altered composition relative to untreated or Bt maize fields (Camerini et al. 2024, Meissle et al. 2022). In a direct field comparison conducted in Italian maize, insecticide application significantly reduced natural parasitism by *Trichogramma brassicae* and disrupted aphid predator community structure, highlighting the broader ecosystem costs of CM even when pest suppression targets are met (Camerini et al. 2024).

It might be expected that the high pheromone concentrations maintained by the dispensers in mating-disruption plots would enhance egg parasitism, because *T. busseolae* is known to use the host sex pheromone as a long-range kairomone for host location (Colazza et al. 1997). The use of host sex pheromone as a foraging kairomone is a widespread phenomenon among *Telenomus* and related scelionid egg parasitoids, and has been documented across multiple tritrophic systems involving Lepidoptera and their egg parasitoids (Hilker and Meiners 2006, Fatouros et al. 2016). The capacity of parasitoids to eavesdrop on host pheromone signals for host location represents an important link between chemical pest management tools and biological control efficacy that deserves further mechanistic investigation in this system. In the present trial, however, this potential benefit was not borne out statistically: with generations pooled, seasonal egg parasitism in the integrated and biotechnical plots (55% and 53% in 2012; 21% and 22% in 2013) did not differ from the UC, and only CM was significantly lower (Table 3). Two considerations further temper a kairomonal interpretation. First, mating-disruption plots contained fewer host egg masses, so an inverse density-dependent response—a higher proportion of a scarcer host population being parasitized—could raise percentage parasitism without any change in parasitoid behavior. Second, with a single unreplicated plot per treatment and only three nested sub-plots, the design cannot test attraction at the field scale. The robust result is therefore that synthetic pyrethroids suppressed parasitism, not that MD enhanced it; the kairomone hypothesis remains speculative and would require a replicated experiment that directly measures parasitoid response to the pheromone source.

Grain yield results were consistent across both years: CM plots produced the highest yields, followed by IM and BTM (which did not differ significantly from each other), with UC lowest. The yield advantage of IM over BTM was not statistically significant in either year, indicating that *T. evanescens* releases did not contribute meaningfully to yield beyond the effect of MD alone. This is not surprising given that *S. nonagrioides—*the heavier yield-affecting pest in this system—was only partially suppressed by MD, while *O. nubilalis* was well controlled by *T. evanescens*. The residual *S. nonagrioides* pressure was apparently sufficient to equalize yield outcomes between IM and BTM. This finding suggests that the primary bottleneck for yield improvement in the integrated approach is more effective suppression *of S. nonagrioides.*

The chemical approach achieved the highest yields but at the cost of disrupting natural enemy populations, as evidenced by reduced parasitism rates in CM plots. The yield gap between CM and IM/BTM plots was ∼100–150 kg/da in both years, representing roughly 15%–25% of the CM yield. Whether this gap can be narrowed by optimizing MD parameters (e.g. dispenser density, timing, inclusion of additional pheromone components) or by supplementing with selective insecticides compatible with *T. evanescens* (such as indoxacarb or chlorantraniliprole) warrants further investigation (Bayram et al. 2010a, Kurtuluş and Kornoşor 2015). Importantly, both indoxacarb and chlorantraniliprole have been classified as harmless or minimally harmful to *Trichogramma* spp. and related scelionid egg parasitoids in IOBC-compliant selectivity assessments, making them promising candidates for integration into augmentative biological control programs where supplementary chemical intervention is deemed necessary (Kurtuluş and Kornoşor 2015, Nozad-Bonab et al. 2021, Exteckoetter et al. 2025). Foliar applications of Bacillus thuringiensis subsp. kurstaki preparations represent another potentially compatible option for a sustainable, nonpyrethroid insecticidal component within this system, and have previously proven effective against ECB when combined with augmentative Trichogramma releases (Burgio and Maini 1995).

Ongoing regional surveys of *S. nonagrioides* pheromone composition are needed to ensure that commercial dispensers remain well matched to local population chemistry, as ­pheromone race variation could explain some of the year-to-year variability in MD efficacy observed in this and other ­studies. Phylogeographic analyses have revealed substantial population-level genetic structuring in *S. nonagrioides* across the Mediterranean basin, with Turkish and Near Eastern populations forming distinct genetic lineages that may differ from Western European reference populations used in commercial pheromone formulation development (Goftishu et al. 2019). Verifying the pheromone blend optimum for Çukurova populations against local electrophysiological and field trapping data would be a valuable step toward maximizing dispenser efficacy in this region.

The design limitations discussed above also point to concrete changes for future trials of this kind. With treatments confined to one large plot per year under natural infestation, location effects arising from the uneven spatial distribution of natural infestation, and possible soil heterogeneity between fields, cannot be fully separated from treatment effects; the joint year-as-factor analysis adopted throughout this revision only partly addresses this, since it can show whether a treatment ranking held across two independently sited trials but cannot substitute for genuine within-year replication. Under natural infestation, we would recommend a randomized complete block design with more than three replicate blocks per treatment per year, which would allow field-position effects to be estimated and removed rather than confounded with treatment. Where sufficient land is not available for such a design, artificial infestation with laboratory-reared egg masses or neonate larvae, released at a standard density across all sub-plots, would achieve a more spatially uniform starting population and reduce the dependence of treatment comparisons on the natural, and often patchy, distribution of the pest. We flag this as a priority for future work in this system rather than a correction that can be made to the present dataset, since it requires a different field layout at the time of establishment.

The results of this study have direct relevance for IPM program development in the Çukurova region and similar Mediterranean maize-growing areas. The current reliance on synthetic pyrethroids across the region imposes substantial selection pressure on target pests. The integrated approach evaluated here—MD for *S. nonagrioides* combined with *T. evanescens* augmentation for *O. nubilalis*—offers a viable, pesticide-free alternative that maintained yields ∼40%–70% above untreated levels, and should be extended to manage newly introduced pests like *Chilo partellus* Swinhoe (Lepidoptera: Crambidae) and *Spodoptera frugiperda* (J.E. Smith) (Lepidoptera: Noctuidae) (Sertkaya et al. 2014, Pehlivan and Atakan, 2022). Compatibility of Bacillus thuringiensis subsp. kurstaki preparations with these newly introduced pests, alongside the biological and biotechnical components evaluated here, should also be assessed as part of a broader, sustainable insect control strategy for the region.

## Data Availability

The data supporting the findings of this study are available from the corresponding author upon reasonable request.
